# Characterization of the inner membrane protein BB0173 from *Borrelia burgdorferi*

**DOI:** 10.1186/s12866-017-1127-y

**Published:** 2017-11-22

**Authors:** Christina M. Brock, Manuel Bañó-Polo, Maria J. Garcia-Murria, Ismael Mingarro, Maria Esteve-Gasent

**Affiliations:** 1 Department of Veterinary Pathobiology, College of Veterinary Medicine and Biomedical Sciences, Texas A&M University, TAMU-4467, College Station, TX 77843 USA; 2 Department of Biochemistry and Molecular Biology, Estructura de Recerca Interdisciplinar en Biotecnologia i Biomedicina (ERI BioTecMed), Faculty of Biology, University of Valencia, E-46100 Burjassot, Valencia Spain; 3 Current affiliation: Department of Entomology, College of Agricultural and Life Sciences, Texas A&M University, College Station, USA

**Keywords:** *Borrelia burgdorferi*, vonWillebrand factor a, MIDAS motif, Transmembrane, Aerotolerance

## Abstract

**Background:**

The bacterial spirochete *Borrelia burgdorferi* is the causative agent of the most commonly reported arthropod-borne illness in the United States, Lyme disease. A family of proteins containing von Willebrand Factor A (VWFA) domains adjacent to a MoxR AAA+ ATPase have been found to be highly conserved in the genus *Borrelia*. Previously, a VWFA domain containing protein of *B. burgdorferi,* BB0172, was determined to be an outer membrane protein capable of binding integrin α3β1. In this study, the characterization of a new VWFA domain containing membrane protein, BB0173, is evaluated in order to define the location and topology of this multi-spanning membrane protein. In addition, functional predictions are made.

**Results:**

Our results show that BB0173, in contrast to BB0172, is an inner membrane protein, in which the VWFA domain is exposed to the periplasmic space. Further, BB0173 was predicted to have an aerotolerance regulator domain, and expression of BB0173 and the surrounding genes was evaluated under aerobic and microaerophilic conditions, revealing that these genes are downregulated under aerobic conditions. Since the VWFA domain containing proteins of *B. burgdorferi* are highly conserved, they are likely required for survival of the pathogen through sensing diverse environmental oxygen conditions.

**Conclusions:**

Presently, the complex mechanisms that *B. burgdorferi* uses to detect and respond to environmental changes are not completely understood. However, studying the mechanisms that allow *B. burgdorferi* to survive in the highly disparate environments of the tick vector and mammalian host could allow for the development of novel methods of preventing acquisition, survival, or transmission of the spirochete. In this regard, a putative membrane protein, BB0173, was characterized. BB0173 was found to be highly conserved across pathogenic *Borrelia,* and additionally contains several truly transmembrane domains, and a *Bacteroides* aerotolerance-like domain. The presence of these functional domains and the highly conserved nature of this protein, strongly suggests a required function of BB0173 in the survival of *B. burgdorferi.*

## Background

Lyme disease (LD) is the most prevalent arthropod borne pathology in the United States, causing illness in more than 30,000 cases annually according to the Centers for Disease Control and Prevention [1]. However, it was also recently reported that the true number of infections may actually approach 300,000 cases per year [2]. The causative agent, *Borrelia burgdorferi*, is transferred via the bite of an infected *Ixodes* tick [3]. Those infected may experience symptoms ranging from *erythema migrans*, malaise, and fever during the first few weeks post tick bite and may progress to facial paralysis, palpitations, and arthritis in the later stages of disease [4]. Due to both the prevalence and multisystemic symptoms of the disease, investigating the biology of *B. burgdorferi* is of utmost importance. By understanding the mechanisms that allow *B. burgdorferi* to live in the highly disparate environments of the tick and mammalian host, novel methods to control the transmission and dissemination of this pathogen may be identified.

It has been shown previously that interactions between spirochaetal and host cells occur during migration of *B. burgdorferi* within the mammalian host away from the tick bite to areas of long term survival, such as the skin, joints, heart and bladder*.* These host-pathogen interactions are required for the pathogenicity of Lyme disease *Borreliae,* particularly as the pathogen responds to changes in temperature, pH, oxygen concentration/availability, and the structural environment through differential gene expression [5–9]*.* With regards to the endothelial cells, *B. burgdorferi* has been shown to bind the extracellular matrix (ECM) components (fibronectin, laminin, collagens (type I, III, and IV)) and integrins, among other components, through *Borrelial* proteins such as BBK32, BBA33, ErpX, P66, BBA07, BB0172, DbpA, DbpB, OspF and Lmp1, respectively [7, 8, 10–20]. Due to their extracellular exposure and relevance for the persistence of the pathogen in the mammalian host, the characterization of these proteins has potential value to the prevention of Lyme disease as drug and vaccine targets.

One approach to identifying these targets is comparative genome analysis. One such analysis performed by Subramanian et al. [21] highlighted conserved genes between the spirochetes *B. burgdorferi* and *Treponema pallidum.* Of the genes highlighted in this analysis, a notable family of genes were reported, the von Willebrand Factor A (VWFA) domain-containing proteins. *In B. burgdorferi,* the VWFA domain-containing proteins BB0172, BB0173, BB0175 and BB0325 are found on the linear chromosome. The presence of the VWFA domain within *Borrelial* proteins is of note because of the known function of this domain in eukaryotes, playing roles in cell adhesion, particularly regarding interactions with extracellular matrix (ECM) components [22]. Additionally, *bb0170* to *bb0176* genes are found to have close similarity to a region of the genome of the aerotolerant anaerobe *Bacteriodes fragilis*, the *BatI* (*B* *acteriodes* aerotolerance) complex [23]. This conserved genomic region has also been described in *Rhizobium leguminosarum* and *Leptopsira interrogans*, although no definite function has been determined [23, 24].

Our lab previously characterized BB0172 protein, which was determined to contain two transmembrane domains and a VWFA domain that is exposed to the host environment [17]. Additionally, it was demonstrated that BB0172 could bind the integrin α_3_β_1_ through the VWFA domain. The *bb0172* gene was also found to be differentially regulated based on the environmental shift between the tick vector and mammalian hosts. Due to the conservation, localization, regulation, and presence of specific protein domains thought to play a role in regulation and adhesion, it was proposed that *bb0172* plays an additional role in the invasion of *B. burgdorferi* within the mammalian host. As such, nearby, similarly structured genes could also be important for host-pathogen interactions. Taking into account the arrangement and predicted function of surrounding genes, *bb0173* is expected to also play a role in host invasion*.*

To better understand the contribution of *bb0170* to *bb0176* open reading frame (ORF) products to the survival and pathogenicity in *B. burgdorferi* and other *Borrelia* species, in the present study we have evaluated the membrane insertion and cellular localization of BB0173 protein. BB0173 is a conserved hypothetical protein that contains several predicted transmembrane regions, a VWFA domain and a metal binding motif. Through evaluation beginning with an in silico analysis and progressing to in vitro DNA and protein studies, the membrane topology of this protein is explored in order to characterize its cellular localization and potential function. Taken together, the investigation of these genes will enhance the understanding of the biology of *B. burgdorferi*, and may lead to an increased ability to target the pathogen using novel therapeutics.

## Results

### Homology of *B. burgdorferi* aerotolerance mediating genes and BB0173 protein features

**Fig. 1:**
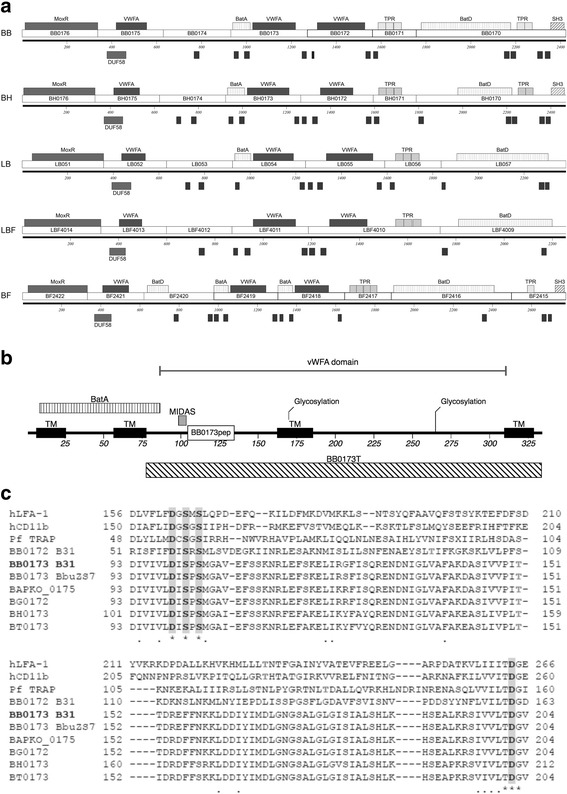
Organization and conservation of *bb0173*. **a** Schematic demonstrating the similarity between the Bat region of *Borrelia burgdorferi* (BB), *Borrelia hermsii* (BH), *Leptospira interrogans* (LB), *Leptospira biflexa* (LBF), and *Bacteroides fragilis* (BF). Note the similarities across BB0172 through BB0176. **b** Map demonstrating the pertinent domains of BB0173. The map demonstrates the three transmembrane domains (amino acids: 7–25, 57–77, 310–328), VWFA domain (amino acids: 87–328), MIDAS motif (amino acids: 99–103), BatA domain (amino acids: 9–86), and N-glycosylation sites (amino acids 170–172, 265–267). Additionally, a 30-mer peptide is denoted, BB0173_pep_, which was used to generate chicken anti-BB0173 antibodies. BB0173_T_, the truncated BB0173 protein, was also used to generate chicken anti-BB0173 antibodies. **c** Clustal W (v1.83) alignment of *B. burgdorferi* B31 BB0173 (bold) against homologues in *B. burgdorferi* ZS7 (BB0173 BbZS7) *Borrelia garinii* (BG0172), *Borrelia afzelii* (BAPKO_0175), and the relapsing fever species *Borrelia hermsii* (BH0173) and *Borrelia turicatae* (BT0173). Alignments are also made to *B. burgdorferi* B31 BB0172 (BB0172 B31), which was found to be very similar in sequence and topology. There is also homology seen to *Plasmodium falciparum* membrane protein TRAP (Pf TRAP) as well as to the human adhesins LFA-1 (hLFA-1) and CD11b (hCD11b). Conserved residues corresponding to the MIDAS motif are highlighted, including the DXSXS as well as the threonine (T) required for MIDAS function

Upon investigating ORFs surrounding *bb0173* on the *B. burgdorferi* linear chromosome, other similarities appeared between *bb0170* to *bb0176* and the equivalent regions of *B. fragilis, R. leguminosarum, L. interrogans, and L. biflexa* [25]*.* The domains are highly conserved in each of these organisms, including the VWFA, Bat, SRC Homology 3 (SH3)_,_ membrane-spanning, tetratricopeptide repeats (TPR), a domain of unknown function (DUF58), and the presence of a MoxR type ATPase (*bb0176*), as seen in Fig. 1a. It has been well documented that MoxR ATPases are found near VWFA domain-containing proteins [26], and has been observed to be the case in the *Borrelia* species evaluated in this paper. Further, *bb0170* appears to be a fusion protein with homology to both *bf2416* and *bf2415* of *Ba. fragilis*, consolidating the BatD, TPR, and SH3 domains into a single protein in *B. burgdorferi* and *B. hermsii (bb0170* and *bh0170*, respectively*)*. Although the *Ba. fragilis* genome encodes duplicates of the BatA (*bf2419* and *bf2418*) and BatD domains (*bf2420* and *bf2416*), these are only predicted to be present in single copy in this region of the genomes of both *Borrelia* and *Leptospira* (Fig. 1a)*.* Interestingly, spirochete *Treponema* has VWFA domain-containing proteins that are much less similar to the VWFA domain-containing proteins of *B. burgdorferi* than organisms in the phylum Proteobacteria [17, 21].

Initially, *bb0173* was predicted to contain four putative transmembrane domains, in addition to the BatA complex and VWFA domain containing a MIDAS (Metal Ion Dependent Adhesion Site) motif. The arrangement of the features present in *bb0173* gene after analysis is schemed in Fig. 1b, showing the predicted transmembrane domains, in addition to the regions utilized to design antigenic peptides to be used for antibody production in a murine model. Moreover, alignments with other VWFA domain-containing proteins make a strong case for a functional MIDAS motif, when comparing conserved residues DXSXS (where X represents any amino acid) and the downstream conserved location of Asp208 residue (T**D**G motif, residues 207–209) known to be required for coordination of metal ions (Fig. 1c).

### Expression of bat-like genes

**Fig. 2:**
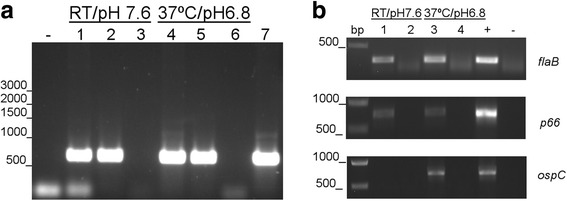
Expression of *bb0173* cDNA upon temperature shift. *B. burgdorferi* B31A3 strain was grown under unfed tick conditions (RT/pH 7.6) to late log phase then shifted to fed tick conditions (37 °C/pH 6.8) before collection of mRNA. The purified mRNA was reverse transcribed to cDNA, and PCR was performed to detect *bb0173, flaB, p66*, and *ospC.* Water was used as a negative control (−). **a** RNA samples were tested for DNA contamination in lanes 3 and 6. Genomic DNA was run in lanes 2 and 5 and served as the positive control. In lanes 1 and 4, cDNA samples were loaded. To confirm functionality of primers, a second genomic DNA sample was applied in lane 7. **b** The same shifting conditions were used to generate DNA samples as previously. cDNA samples are in lanes 1 and 3, RNA in lanes 2 and 4, and genomic DNA is labeled as (+). Negative control is water, as above. On the left of the figure, the DNA ladder is shown and sizes are denoted in basepairs

Prior to the characterization of BB0173 protein, the conditions at which *bb0173* gene is expressed in *B. burgdorferi* were evaluated*.* From the cellular mRNA levels, we evaluated the expression of *bb0173* under unfed tick growth conditions (25 °C and pH 7.6) as well as during conditions shifted to fed-tick conditions (37 °C and pH 6.8), as previously described [17]. As shown in Fig. 2a, the expression of *bb0173* was detected at both unfed as well as during fed-tick conditions. Controls for the PCR are shown in Fig. 2b. The differentially regulated *ospC* is expressed only during fed-tick conditions, and the constitutively expressed *flaB* and *p66* are present under both unfed and fed tick conditions, as expected.

**Fig. 3:**
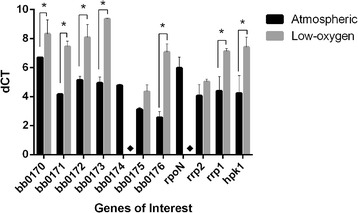
Expression of *bb0170* to *bb0176* under decreased-oxygen conditions. Gene expression of the Bat-like genes is quantified under atmospheric oxygen and decreased oxygen conditions. The expression of each gene is determined by comparing the expression under atmospheric oxygen to the expression under low-oxygen growth conditions after normalization to the endogenous control gene, *flaB* (ΔCT = Ct (goi) – Ct (ref); where “goi” refers to the gene of interest, and “ref” to the reference gene used in the study)*.* All genes tested were found to increase expression under low-oxygen conditions except for *bb0174* and *rpoN*, which both became undetectable in the low-oxygen condition, as denoted by the diamond (♦). For this experiment, *rpoN, rrp1, hpk1,* and *rrp2* have been included as control genes. Statistical analysis was performed using the Holm-Sidak multiple comparisons test with a 95% confidence interval. Each gene was evaluated in triplicate. *Denotes statistical differences: *P value <0.05, **P value <0.01, ***P value <0.001 and ****P value <0.0001

Due to the prediction of a BatA domain, evaluation of oxygen levels on gene transcripts was performed. *B. burgdorferi* cultures were grown under standard (atmospheric) or low oxygen conditions, and *bb0170* to *bb0176* were evaluated for gene expression changes between the two environments. In order to quantify these changes, q-RT-PCR analysis was performed to evaluate relative expression of *bb0170* to *bb0176* (Fig. 3). From this analysis, it was determined that each of these genes displayed a decrease in expression level under the low-oxygen growth condition. Intriguingly, *bb0174* and *rpoN* gene expression was decreased to undetectable levels when grown under low-oxygen conditions. Furthermore, of *bb0173* and *bb0176* expression was severely reduced, more than 20-fold when compared to growth under aerobic (atmospheric) conditions. Significant decreases in expression between aerobic and low-oxygen growth conditions were also seen for *bb0170, bb0171, bb0172,* and for control genes*, rrp1,* and *hpk1.* Gene bb0175, as well as control gene *rrp2,* expressed similarly on both growth conditions*.*

### Insertion of BB0173 hydrophobic regions into ER-derived microsomal membranes

**Fig. 4:**
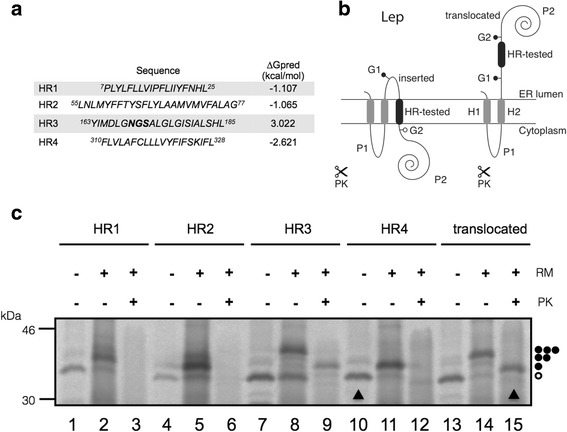
Insertion of hydrophobic regions of BB0173 into membranes using Lep as model protein. **a** The HR sequence in each construct is shown together with the predicted G apparent value, which was estimated using the ∆G prediction algorithm available on the Internet (http://dgpred.cbr.su.se/). Glycosylation acceptor site is shown in bold. **b** Schematic representation of the Lep construct used to report insertion of hydrophobic regions of BB0173 into endoplasmic reticulum membranes. The TM segment under investigation (HR-tested) was introduced into the P2 domain of Lep, flanked by two artificial glycosylation acceptor sites (G1 and G2). Recognition of the tested sequence as a TM domain by the translocon machinery results in the location of only G1 in the luminal side of the ER membrane, preventing G2 glycosylation (left). The Lep chimera will be doubly glycosylated when the sequence being tested is translocated into the lumen of the microsomes (right). **c** In vitro translation in the presence of membranes of the different Lep constructs. Constructs containing HR1 (residues 7 to 25; lanes 1–3), HR2 (residues 55 to 77; lanes 4–6), HR3 (residues 163 to 185; lanes 7–9) and HR4 (residues 310 to 328; lines 10–12) were translated in the presence (+) and absence (−) of rough microsomes (RM) and proteinase K (PK). Bands of non- glycosylated proteins are indicated by a white dot; singly and doubly glycosylated proteins are indicated by one and two black dots, respectively. In the case of Lep-HR3 construct a triply glycosylated band was observed (lane 8) due to the presence of an acceptor NGS site (residues 169–171) within the (translocated) hydrophobic region. The protected doubly-glycosylated H2/HR3/P2 fragment is indicated by an arrowhead. Control HRs were used to verify sequence translocation (translocated; lanes 13–15)

BB0173 is predicted to be a membrane protein, therefore, to identify the presence of putative hydrophobic regions (HRs) BB0173 amino acid sequence was parsed to test the performance of the ΔG Prediction Server (http://dgpred.cbr.su.se/). Given the amino acid sequence, this algorithm predicts the corresponding apparent free energy difference, Δ*G*_app_, for insertion of each hydrophobic region into the endoplasmic reticulum (ER) membrane by means of the Sec61 translocon [27, 28]. Figure 4a shows the predicted Δ*G*_app_ values for the hydrophobic regions detected. The negative Δ*G*_app_ values for the HR1, HR2 and HR4 regions predict a transmembrane (TM) disposition, whereas the positive value computed for HR3 predicts that this sequence does not integrate into ER membrane.

To test these predictions, we assayed the membrane insertion capabilities of these HRs using an in vitro experimental system based on the *Escherichia coli* inner membrane protein leader peptidase (Lep) [28, 29], which accurately determines the integration of TM segments into microsomal membranes. Lep consists of two TM segments (H1 and H2) connected by a cytoplasmic loop (P1) and a large C-terminal domain (P2) (Fig. 4b), and inserts into ER-derived rough microsomal membranes (RMs) with both termini located in the lumen. The analyzed segment (HR tested) is engineered into the luminal P2 domain and is flanked by two acceptor sites (G1 and G2) for *N*-linked glycosylation. Single glycosylation (i.e., membrane integration) results in a molecular mass increase of 2.5 kDa relative to the observed molecular mass of Lep expressed in the absence of microsomes (Fig. 4b, left). A molecular mass shift of 5 kDa occurs upon double glycosylation (i.e., membrane translocation of the HR-tested) (Fig. 4b, right). This system has the obvious advantage that the insertion assays are performed in the context of a biological membrane.

Translation of Lep chimeric constructs harbouring the BB0173 regions predicted by the Δ*G* Prediction Server (Fig. 4a) resulted mainly in single-glycosylated forms for HR1, HR2 and HR4 regions (Fig. 4c, lanes 2, 5 and 11), except for HR3 containing construct (Fig. 4c, lane 8). Interestingly, in this latter case, translation products were found mostly triple-glycosylated. It should be mentioned that BB0173 sequence includes a native potential *N*-glycosylation site at Asn187, i.e. within HR3 region (see Fig. 4a), adding an *N*-glycosylation motif that would be modified only if this region is not inserted into the lipid bilayer (Fig. 4c, lane 8). These results were confirmed by proteinase K (PK) treatment. Digestion with PK degrades membrane protein domains located exclusively towards the cytosol, while membrane-embedded or lumenally exposed domains are protected. As expected, Lep chimeras bearing HR1, HR2 and HR4 regions were sensitive to PK digestion (Fig. 4c, lanes 3, 6 and 12). However, Lep constructs containing HR3 sequence were partially resistant to the protease treatment due to its luminal P2 localization (Fig. 4c, lane 9, arrowhead, expected size ≈33.5 kDa).

### Membrane insertion and topology of BB0173 into the ER membrane

**Fig. 5:**
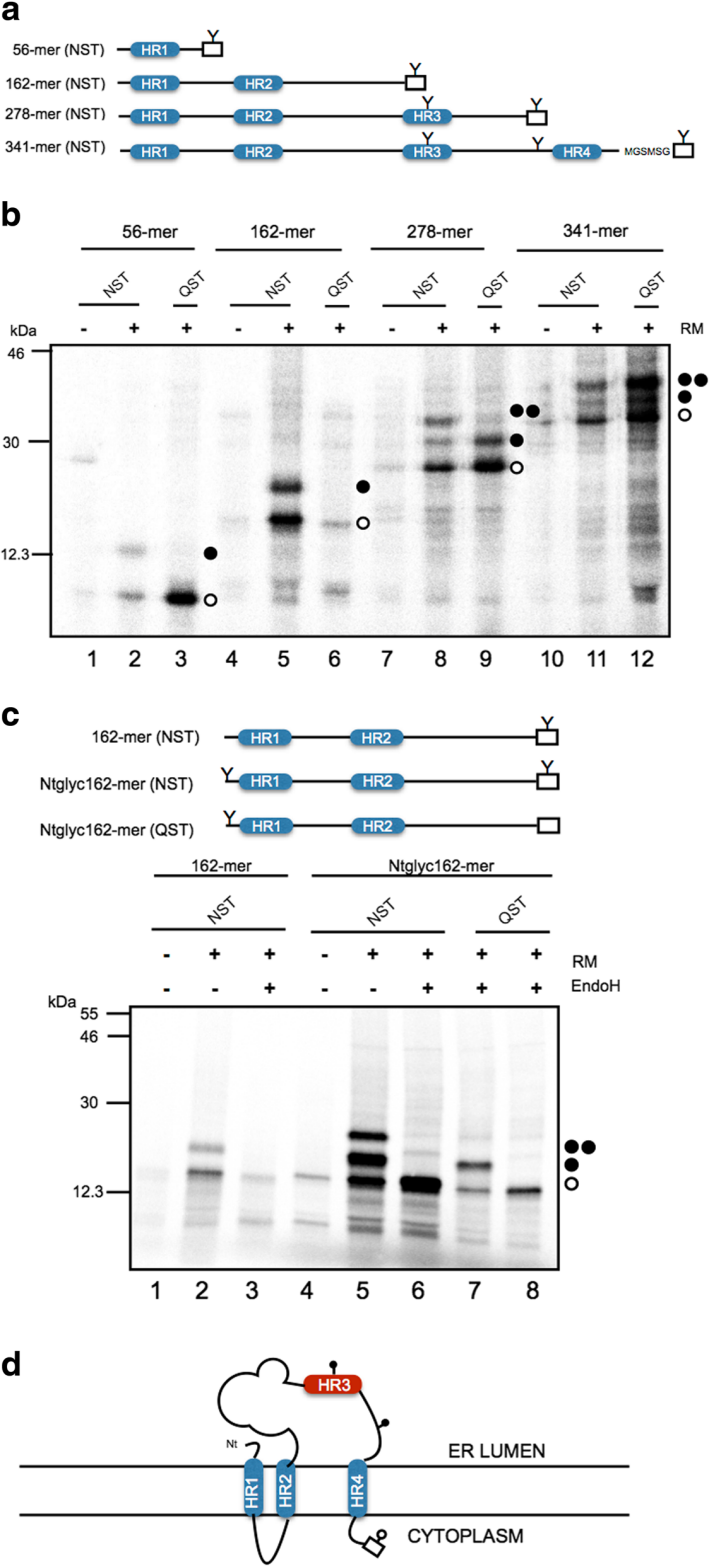
In vitro analysis of truncated BB0173 constructs. To monitor the membrane orientation of truncated BB0173 molecules a glycosylatable (NSTMSM) tag (white rectangle) was added at position 56 (56mer), 162 (162mer), 278 (278 mer) and 341 (341mer). **a** Schematic representation of the constructs used in the assay. The position of the glycosylation sites is marked with a Y symbol. The presence of a TM segment identified by the ΔG prediction server (http://dgpred.cbr.su.se/) in each construct and the required linker sequence preceding the glycosylatable tag to allow glycosylation is also included for 341mer truncates. **b** In vitro translation of the 56mer, 162mer, 278mer and 341mer truncates in the presence (+) or absence (−) of rough microsomes (RM). A white dot marks the non-glycosylated form of the protein while a black dot indicates glycosylation of the C-terminal tag. **c** In vitro translations in the presence or absence of RM of 162mer truncated constructs were performed bearing an acceptor (NST) or non-acceptor (QST) at N-terminal and/or C-terminal glycosylation tag. White and black dots indicate non-glycosylated and glycosylated molecules respectively, as in panel **b**. **d** Schematic representation of the membrane topology of 341mer truncates. A hydrophobic region is noted as a blue box when inserted in the membrane, or as a red box if it is not recognized by the translocon as a TM domain. The position of the glycosylatable tag (white rectangle) and its glycosylation status (white and black dots, represents non-glycosylated and glycosylated respectively) is also shown

To experimentally map the membrane insertion and topology of BB0173 protein, we prepared a series of polypeptide truncates containing an added C-terminal glycosylation tag (Asn-Ser-Thr, NST), which has been proven to be efficiently modified in the in vitro translation system [30–32]. The constructs used are delineated in Fig. 5a. As shown in Fig. 5b, translation products containing the N-terminal 56 residues of BB0173 sequence, including the first predicted TM segment (HR1) plus an optimized glycosylatable C-terminal tag (56-mer NST), were singly-glycosylated in the presence of microsomal membranes (Fig. 5b, lane 2). The nature of these higher molecular weight polypeptide species was analysed by translating the first 56 residues with a C-terminal tag that includes a non-acceptor (Gln-Ser-Thr) site for N-glycosylation (56-mer QST), ensuing the elimination of the higher molecular mass band (Fig. 5b, lane 3), confirming the sugar source of their retarded electrophoretic mobility and suggesting the 56mer polypeptide insertion into the microsomal membrane with an N-terminal cytoplasmic orientation. The low glycosylation efficiency (26 ± 3%) observed for this truncated (56-mer) protein, suggests either a rather inefficient targeting to the membrane or the coexistence of two different topologies for these short truncated molecules.

Truncated 162-mer polypeptides, which include the first two HRs (Fig. 5b, lanes 4–6), were efficiently glycosylated (45 ± 4% of glycosylation, lane 5), denoting carboxyl terminal (Ct) translocation. To ensure proper insertion of both HRs as a hairpin, an additional N-glycosylation acceptor site was introduced at the N-terminus of BB0173 (insertion NST at position 2, K2 N), creating the construct NtglycBB0173. Translation of Ct tagged 162-mer NtglycBB0173 yielding singly- and doubly-glycosylated forms (Fig. 5c, lane 5). The presence of double-glycosylated forms suggests the translocation of both N- and C- termini (Fig. 5c). When a non-acceptor (QST) site as C-terminal tag was used, a singly-glycosylated form was observed (Fig. 5c, lane 7), consistent with the N-terminal translocation of the polypeptide chain.

The putative insertion of the third predicted TM segment (HR3) was tested by translating a 278-residue truncation with the same C-terminal glycosylatable tag (278-mer). As mentioned before, wild type BB0173 carries a potential glycosylation site at Asn187 (see Fig. 5a). In case of translocation across the microsomal membrane, both Asn187 and the added C-terminal glycosylation tag should be modified rendering doubly-glycosylated forms. Translation of construct 278-mer produced mainly doubly-glycosylated (59%) forms, indicating that HR3 is predominantly translocated (Fig. 5b, lane 8). When the same chimera was translated with a C-terminal tag harbouring a non-acceptor site (QST), only singly glycosylated forms were detected (Fig. 5b, lane 11), corresponding to native Asn187 modification.

Finally, the insertion of the predicted HR4 in its natural context was analyzed by translating full-length *bb0173* gene (341-mer). It should be noted that wild type BB0173 sequence carries a second glycosylation site at Asn273 (see Fig. 5a). Translation of C-terminal tagged full-length constructs either with an acceptor (NST) or a non-acceptor (QST) glycosylation sites produced double glycosylated forms (Fig. 5b, lanes 11 and 12), indicating that HR4 is efficiently inserted. Overall, these results evidenced that BB0173 protein inserts into the ER membrane with Nt-lumenal/Ct-cytosol orientation, where HR1, HR2 and HR4 behave as truly transmembrane segments, and translocate the loop between HR2 and HR4, which contains the VWFA domain (Fig. 5d).

### Cellular localization of BB0173 within *B. burgdorferi*

With combined knowledge, both from membrane insertion data and expression conditions, we evaluated the actual cellular localization of BB0173 within *B. burgdorferi* cells using both a protease protection assay and detergent phase separation assay.

**Fig. 6:**
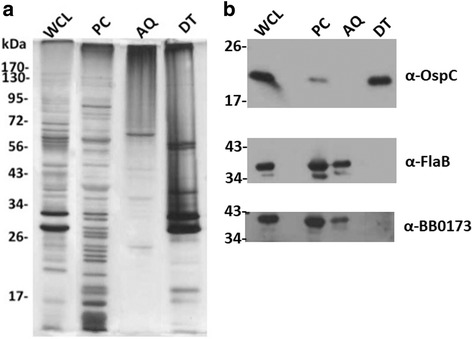
Localization of BB0173 to the aqueous and inner membrane fractions after treatment with detergent. *B. burgdorferi* cells disrupted using the detergent Triton X-114 separated into three distinct fractions, the aqueous (AQ), protoplasmic cylinders (PC), and detergent (DT) phases. The phases were separated using SDS-12% PAGE and either stained using Silver Stain Plus (Biorad, Hercules, CA) (**a**) or were transferred to a PVDF membrane and probed using anti-BB0173_T_ and a secondary anti-chicken HRP-conjugated antibody Lane 1 is *B. burgdorferi* whole cell lysate. Lane 2 is AQ, Lane 3 is PC, and Lane 4 is DT (**b**). Controls for outer membrane and inner membrane proteins were OspC and FlaB

The Triton X-114 detergent phase separation assay evaluated localization of proteins within the cell to either the inner or outer membrane or to the cytoplasm. This separation method was used to evaluate BB0173 localization from cells grown at 32 °C/pH 7.6, which were then silver stained to evaluate equal loading (Fig. 6a). Using Western Blot with chicken-anti-BB0173, the protein was detected in both the aqueous (AQ) phase and protoplasmic cylinders (PC), and no band was seen in the detergent (DT) phase (Fig. 6b). Control proteins treated in parallel were observed in the expected fractions based on their described localization (Fig. 6b) including: OspC (DT and PC) and FlaB (AQ and PC). Therefore, these results suggest that BB0173 is associated with the inner membrane of *B. burgdorferi*.

**Fig. 7:**
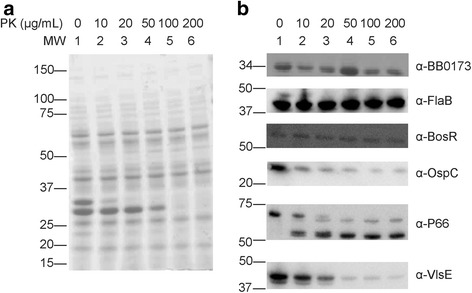
Protection of BB0173 from protease degradation. Surface proteins of *B. burgdorferi* are degraded by serine protease Proteinase K (PK). Whole cell lysates were treated with doses ranging from 0 to 200 μg/mL PK prior to separation using SDS-12% PAGE. Gels were either visualized using Coomassie blue staining (**a**) or transferred to a PVDF membrane and probed with antibodies (**b**). BB0173 was detected using anti-BB0173_pep_ and anti-chicken HRP-conjugated antibody. Controls for PK mediated degradation and cell integrity during treatment included intercellular protein BosR and periplasmic protein FlaB, as well as outer membrane proteins OspC, VlsE, and P66

In order to confirm the intracellular localization of BB0173, degradation of extracellularly-exposed proteins of *B. burgdorferi* was performed by proteinase K (PK) treatment. After treating *B. burgdorferi* cells grown at both 25 °C/pH 6.8 and 37 °C/pH 7.6 with PK at a concentration of 200 μg/mL, no apparent change in size was observed in the band corresponding to BB0173, nor did any smaller bands become apparent. To ensure more accurate results, a titration of PK concentration ranging from 0 to 200 μg/mL was used to treat *B. burgdorferi* cells. Samples were coommassie blue stained for equal loading (Fig. 7a) prior to blotting for proteins (Fig. 7b). In each experiment, regardless of concentration of PK used, bands corresponding to OM anchored proteins P66, OspC and VlsE observed a decrease in visualization with treatment. Moreover, periplasmic FlaB and intracellular BosR proteins were unaffected. As mentioned above, BB0173 was seen to be unaffected by protease treatment suggesting an inner membrane location.

## Discussion

**Fig. 8:**
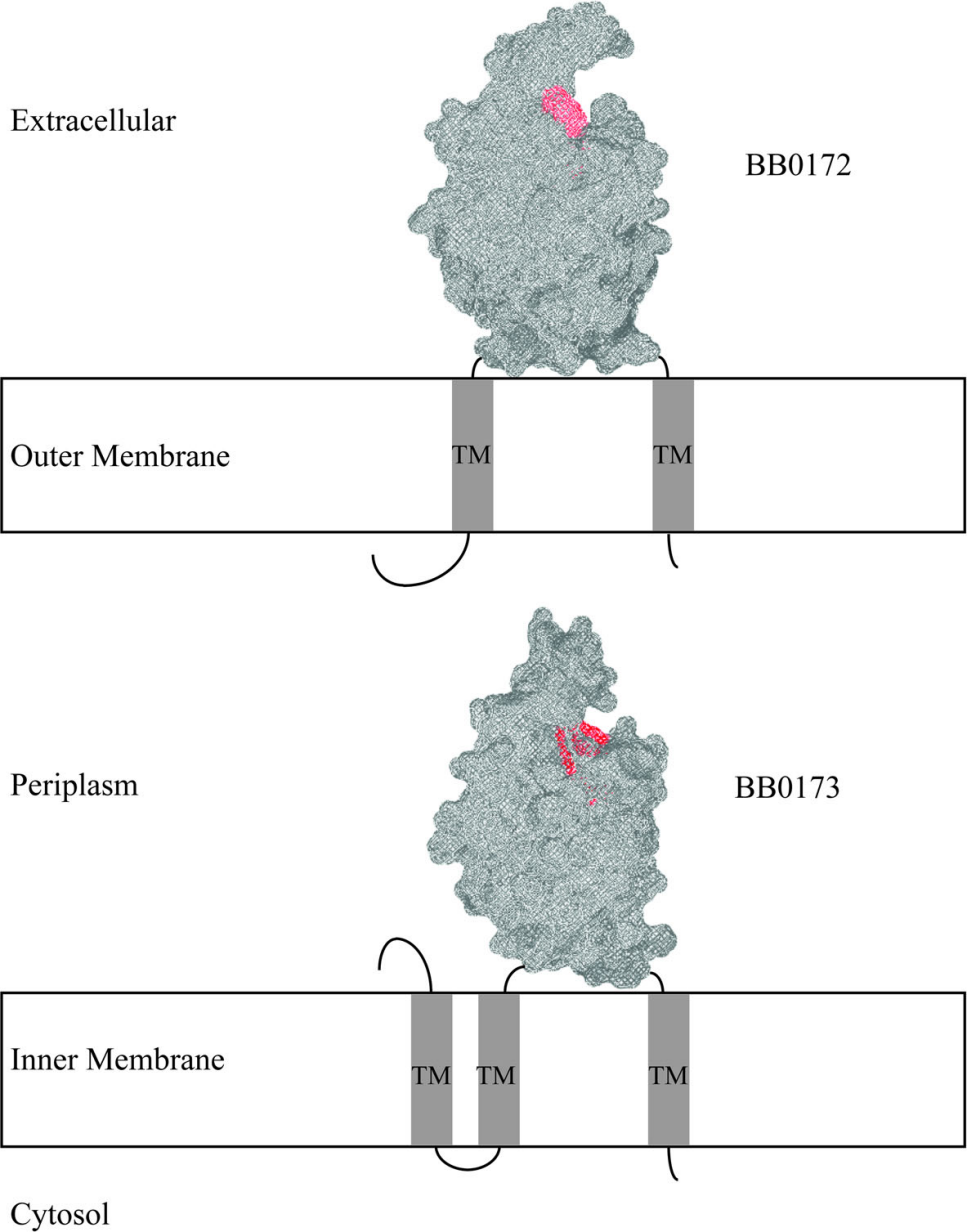
Localization of the tertiary structures of BB0172 and BB0173 within *B. burgdorferi*. Models of the tertiary structures of BB0172 and BB0173 were generated and superimposed onto either the inner or outer membrane as predicted from localization studies

On the *B. burgdorferi* linear chromosome, *bb0172, bb0173, bb0175,* and *bb0325* were identified as genes encoding for proteins containing VWFA domains. Within *B. burgdorferi*, BB0173 is only the second VWFA domain-containing protein to be characterized, the other being BB0172 [17]. Based on the similarity between predicted motifs of BB0172 and BB0173, expression, localization, and ultimately functions were expected to be related. However, our studies revealed that there are key differences between them. BB0172, was determined to be a VWFA domain-containing outer membrane protein with extracellular exposure [17]. In contrast to BB0172, we demonstrated that BB0173 is anchored to the bacterial inner membrane through three TM helices. The topology observed is compatible with having the VWFA domain oriented toward the periplasm, where can interact with both periplasmic and/or outer membrane bound components (see Fig. 8 for a scheme).

The potential role of BB0173 is definitively unique from BB0172, which functions through the binding of mammalian integrin α_3_β_1_ [17]. However, the localization of BB0173 strictly within the cell coupled with the extracellular function of BB0172 could indicate a function as a sensing system. The MIDAS motif is predicted to be accessible to interaction partners in both cases, albeit to the host environment in the case of BB0172 and to the periplasm of *B. burgdorferi* in the case of BB0173. Partial support for this idea comes from the presence of the TPR-like sequences found in neighbor BB0170 and BB0171, which have been suggested*.* to play a role in protein complexing of the BatI-like proteins [25, 33].

In a study by Rogers et al. [34], expression of *bb0170, bb0173, bb0174,* and *bb0175* was found to be increased in *B. burgdorferi* expressing *rrp1* gene compared to bacteria lacking *rrp1.* This increase was especially relevant for *bb0170* and *bb0173* genes [34]. Expression of *bb0174* and *bb0175* increased in *B. burgdorferi* expressing *rrp1* as well, but to a much lower level*.* In the present study, expression of *rrp1* and *hpk1* genes were shown to be significantly decreased in cultures grown at low-oxygen conditions. As such, should the expression of *bb0173* be under control of *hpk1* and *rrp1*, expression of *bb0173* as well as *bb0170, bb0174,* and *bb0175* would decrease as well. Of the genes highlighted by Rogers et al. [34], *bb0173* displayed an important expression decrease under low-oxygen conditions (21.3 fold), while *bb0170* expression decreased by only 3.09 fold, suggesting that while *rrp1* may be involved in regulation of these genes, oxygen mediated gene expression may have additional control mechanisms. Interestingly, *bb0176* was not found to be differentially regulated between *rrp1* according to knockdown data [34]; however, between aerobic and low-oxygen conditions, *bb0176* was the most strongly downregulated target in our study, with a 22.8 fold decrease in expression.

In studies evaluating the effect of temperature on gene expression in *B. burgdorferi*, *bb0175* was found to be highly upregulated when cultivated at 35 °C rather than 23 °C [34–36]*.* Further, *bb0175* expression was shown to be minimally altered in the presence or absence of *rrp1* [34]*.* Our results showing only a modest decrease in *bb0175* expression (2.3 fold) under changing oxygen conditions support the hypothesis that control of *bb0175* transcript expression appears to be mainly directed by temperature conditions.

*BatI*-like genes have also been noted in other spirochetes, such as *Leptospira interrogans* and *Treponema denticoloa* [21, 23]. In each of these cases as well as in *B. burgdorferi*, the VWFA domain containing proteins are found to be associated with a methanol dehydrogenase regulatory (MoxR) ATPase Associated with diverse cellular Activities (AAA). These genes have been suggested to be organized as an operon system, although there is no clear function associated to the proteins they encode [23–25, 37]. In *Rhizobium leguminosarum*, it has been observed that cells with defects in these genes demonstrated envelope and cell morphology changes. This operon is referred to as complex media growth deficient (*cmdA-cmdD*) [24, 38]*.* It is interesting to note that both *R. leguminosarum* and *B. burgdorferi* encounter stark changes of environment, with both between conditions of higher oxygen conditions (*Rhizobium:* free living, *Borrelia*: host) to decreased oxygen conditions (*Rhizobium*: plant host, *Borrelia*: unfed tick) [24, 38, 39]. Conservation of these genes between such diverse species therefore may be due to the survival needs of these bacteria to changing environmental conditions.

Two hypotheses have been addressed in the literature for the purpose of *bb0170* to *bb0176* homologs. One possibility, proposed in relation to both *B. fragilis* and *L. biflexa*, is the potential for these genes to protect the organisms from oxidative stress [23, 25]. This idea is particularly attractive in *B. burgdorferi*, which lacks traditional mechanisms to combat oxidative stress [40]. Taking into consideration the ability to differentially regulate protein expression, namely BB0172, and the lack of mechanisms to deal with oxidative stress, it is plausible that these proteins work together to subdue effects of oxidative damage by generating a periplasmic environment rich in reducing power, although this hypothesis has not been directly proven to be the case neither in *Bacteroides n*or *Leptospira* [23, 25].

The second predicted function for these genes was based on mutations of these genes, which produced defects in envelope integrity when *R. leguminosarum* cells were stressed [24]. In relation to *B. burgdorferi*, this idea may also be a feasible function of the gene complex. Compared to *Escherichia coli* or other gram-negative organisms, the cell wall of *B. burgdorferi* lacks LPS, thus potentially allowing the membrane to be more sensitive to physical stressors [41]. As such, these genes may play a role in supporting cell wall function, particularly in the tick during the blood meal or mammalian infection. As the tick vector engorges, the spirochete must survive a drastic increase in pressure before migrating to the salivary glands and being transmitted to the host [42].

## Conclusions

Taken together, the expression of *bb0173* was regulated by the presence of oxygen, while temperature, pH, or the combination of both had limited effect in regulating its expression. Consequently, in this study we propose that BB0173 is part of an aerotolerance complex similar to that previously described in *Bacterioides*. Furthermore, we demonstrate that BB0173 is an inner membrane protein in contrast with BB0172, which localizes in the outer membrane and binds to host integrins [17]. Consequently, further efforts should be directed to determine the potential function of BB0173 and the aerotolerance gene complex described in this manuscript and by others [23, 24, 38, 43].

The evaluation of hypothetical proteins of organisms such as *B. burgdorferi*, pathogens that are presently not well understood, is a worthwhile endeavor to identify potential targets for diagnostics, prevention, and treatment of disease. Lyme disease is particularly important, as missing the window for treatment can cause a lifetime of ongoing symptoms and prevention or enhanced treatment could change the outcome for these individuals [44, 45]. Specially, due to their highly-conserved nature, elucidation of the function of the *bb0170* to *bb0176* genes set transcends spirochete biology, and can apply broadly to a wide range of bacteria. Conservation of these genes across such a wide variety of bacteria implies that they likely impart a crucial function for survival. Hence, an understanding of the roles of such proteins may facilitate enhanced detection, prevention, and treatment options for Lyme disease as well as other infectious diseases.

## Methods

### Growth conditions of *Borrelia burgdorferi*

DNA used for experiments was extracted from *Borrelia burgdorferi* B31 A3 strain (Table 1). Cells were grown in BSK II with 6% inactivated normal rabbit serum (iNRS) and 1% CO_2_ at either pH 6.8 and 37 °C (fed tick, 37 °C/pH 6.8) or pH 7.6 and 25 °C (unfed tick, 25 °C/pH 7.6). Additionally, *B. burgdorferi* grown under shifted conditions between unfed and fed tick were started under unfed tick growth conditions [17]. Once the culture reaches a density of 2–3 × 10^7^ spirochetes/ml a subculture is started and grown under fed tick conditions until reach a density of 5 × 10^7^ spirochetes/ml. Low oxygen conditions were generated using Oxyrase® for Broth (Mansfield, Ohio) at 0.025 mL Oxyrase® per 1.0 mL BSK II media, as described previously [39]. If no growth condition was specified, cells were grown in BSK II pH 7.6 at 32 °C with 6% iNRS and 1% CO_2_.

### RNA and genomic DNA purification for detecting *bb0173* transcript by PCR

RNA was extracted as previously described [17, 46, 47]. Briefly, *B. burgdorferi* cultures were grown to a density of 2–3 × 10^7^ spirochetes/ml under the shifting conditions outlined above. RNA was extracted by re-suspending the bacterial pellets with 0.2 ml RNA-Bee (Tel-Test, Inc., Friendswood, TX) for every 10^6^ cells. Following extraction with chloroform, RNA was precipitated with isopropanol, washed with 75% ethanol, air dried, and re-suspended in RNase-free water. To remove contaminating DNA, the RNA was treated twice with DNase I at 37 °C for 45 min. Then the total RNA was quantified spectrophotometrically, and reverse transcribed to cDNA using TaqMan reverse transcription reagents (Applied Biosystems, Foster City, CA). From *B. burgdorferi* cultures growing under tick-feeding conditions (37 °C/pH 6.8) and regular growing condition (32 °C/pH 7.6), genomic DNA was obtained by general phenol:chloroform extraction.

### Gene expression

RNA, cDNA, and genomic DNA (positive control) samples from each growing condition were used to detect when *bb0173* was expressed. A 501-bp fragment of *bb0173* was amplified using primers BB0173cDNA-F (*B. burgdorferi* nucleotides 175,811–175,860 and BB0173cDNA-R (*B. burgdorferi* nucleotides 175,334–175,357) (Table 2). Primers specific to the *flaB, ospC,* and *p66* genes were also included as controls for the temperature and pH shift as previously described [17, 48, 49]. PCR products were separated on 0.8% agarose gels and imaged using the Bio-Rad Gel Doc™ XR system.

**Table 2:** Oligonucleotide primers used in this study

Primer pair	RS	Sequence (5’➔3′)	Application
bb0173_T_-NdeI-F	NdeI	ACG CCA TAT GGC TTT AGC AGG TCC TTC	Amplification of *bb0173* for cloning into pCR2.1 and expression vector pET23a
bb0173_T_-XhoI-R	XhoI	ACG CCT CGA GTA GTA TCT CTT TTA AG	
bb0173cDNA-F(nt249–272)		GAA GAT GAT ACA TCT TAG TGC TGG	Amplification of bb0173 cDNA from RNA
bb0173cDNA-R(nt725–749)		CTT CCC TGA TAA AAT TTT CCA GAT	
bb0173TM1-SpeI-F	SpeI	ACG CAC TAG TGG AGG ACC AGG AAA TGA GCC TTT ATA TTT G	Amplification of the first putative *bb0173* transmembrane sequence in frame with the Lep construct
bb0173TM1-KpnI-R	KpnI	ACG CGG TAC CCC TCC TGG TCC CTT TAT CTT GCC TCC TCT	
bb0173TM1–2-SpeI-F	SpeI	ACG CAC TAG TGG AGG ACC AGG AGA TTA TAG ATT AA TTT G	Amplification of the second putative *bb0173* transmembrane sequence in frame with the Lep construct
bb0173TM2-KpnI-R	KpnI	ACG CGG TAC CCC TCC TGG TCC AAC TGA AGG ACC TGC TAA AGC	
bb0173TM2b-SpeI-F	SpeI	ACG CAC TAG TGG AGG ACC AGG ACT AGA TGA TAT TTA TAT TAT G	Amplification of the third putative *bb0173* transmembrane sequence in frame with the Lep construct
bb0173TM2b-KpnI-R	KpnI	AGC CGG TAC CCC TCC TGG TCC AGC CTC AGA ATG CTT TAA ATG	
bb0173TM3-SpeI-F	SpeI	ACG CAC TAG TGG AGG ACC AGG AGA TAT TTA TAA AGA ATT TTT AG	Amplification of the fourth putative *bb0173* transmembrane sequence in frame with the Lep construct
bb0173TM3-KpnI-R	KpnI	ACG CGG TAC CCC TCC TGG TCC CTC TTT TAA GAA AAT TTT TG	
bb0173-NcoI-F.1	NcoI	ACG CCC ATG GAT GTT AAC ATT TAA TGA G	Common amplification start site for truncated insertion constructs
T7BB173		ATA GTA TAA TAC GAC TCA CTA TAG GAA ACC ACC ATG GGC ATG TTA ACA TTT AAT G	Amplification of BB173 with T7 promoter
TM1 NST BB173		TTA TCA GGA CAT CAT GGT GCT GTT ATA ATC CTT AAG TTT TAA	Amplification of BB173 56mer adding C-tag NST
TM1 QST BB173		TTA TCA GGA CAT CAT GGT GCT CTG ATA ATC CTT AAG TTT TAA	Amplification of BB173 56mer adding C-tag QSTMMS
TM2 NST BB173		TTA TCA GGA CAT CAT GGT GCT GTT AAT ATC ATC TAG CTT TT	Amplification of BB173 162mer adding C-tag NST
TM2 QST BB173		TTA TCA GGA CAT CAT GGT GCT CTG AAT ATC ATC TAG CTT TT	Amplification of BB173 162mer adding C-tag QSTMMS
TM3 NST BB173		TTA TCA GGA CAT CAT GGT GCT ATT AAG CAT ACT AGG ATC	Amplification of BB173 278mer adding C-tag NST
TM3 QST BB173		TTA TCA GGA CAT CAT GGT GCT CTG AAG CAT ACT AGG ATC	Amplification of BB173 278mer adding C-tag QSTMMS
TM 4 NST BB173		TTA TCA GGA CAT CAT GGT GCT GTT CCC GGA CAT GCT GCC CAT TAG TAT CTC TTT TAA GAA	Amplification of BB173 341-mer (full length) adding C-tag NST
TM 4 QST BB173		TTA TCA GGA CAT CAT GGT GCT CTG CCC GGA CAT GCT GCC CAT TAG TAT CTC TTT TAA GA	Amplification of BB173 341-mer (full length) adding C-tag QSTMMS
TM2 NST BB173		TTA TCA GGA CAT CAT GGT GCT GTT AAT ATC ATC TAG CTT TT	Amplification of BB173 162mer adding C-tag NST
TM2 QST BB173		TTA TCA GGA CAT CAT GGT GCT CTG AAT ATC ATC TAG CTT TT	Amplification of BB173 162mer adding C-tag QSTMMS
TM3 NST BB173		TTA TCA GGA CAT CAT GGT GCT ATT AAG CAT ACT AGG ATC	Amplification of BB173 278mer adding C-tag NST
TM3 QST BB173		TTA TCA GGA CAT CAT GGT GCT CTG AAG CAT ACT AGG ATC	Amplification of BB173 278mer adding C-tag QSTMMS
bb0170 qPCR F		GTT AAA CCG ATT CCT GGA GAG	Amplification of *bb0170* from cDNA for expression studies
bb0170 qPCR R		CAG CCA AAA CTT GAT GCT GC	
bb0171 qPCR F		AAA TCC ATG TCT TTA ATG	Amplification of *bb0171* from cDNA for expression studies
bb0171 qPCR R		AAC CCT CTC AAG ATT TTC	
bb0172 qPCR F		TAT GGG GAC AAT TCT TAT ATT CAA	Amplification of *bb0172* from cDNA for expression studies
bb0172 qPCR R		CAA TCC CAA CCA CAA AAC TTT CCA	
bb0173 qPCR F		ATT TAA TGA GCC TTT ATA TTT GTT TTT A	Amplification of *bb0173* from cDNA for expression studies
bb0173 qPCR R		GAT CCA TAA TAT AAA TAT CAT CTA GCT T	
bb0174 qPCR F		GAT GGT GAA GAG TTT TCC	Amplification of *bb0174* from cDNA for expression studies
bb0174 qPCR R		TTC TGT TGT AGT GAT TGC	
bb0175 qPCR F		TTT CAT GAG TTT AGG CCG	Amplification of *bb0175* from cDNA for expression studies
bb0175 qPCR R		TGT TGA CTT GCT AAA CCC	
bb0176 qPCR F		TTA CTT GAA GGG GTT CCG	Amplification of *bb0176* from cDNA for expression studies
bb0176 qPCR R		ATC CCT TTC ACG GAG TGC	
rpoN qPCR F		TTG TAC CCC ATT CGC ATT TT	Amplification of *rpoN* from cDNA for expression studies
rpoN qPCR R		GTG AAA ACC CCC AAA AAC AA	
rpoS qPCR F		TTG GGC GAT TTT TCT TCT TC	Amplification of *rpoS* from cDNA for expression studies
rpoS qPCR R		TGC GGG TAA AGG GTT AAA AA	
rrp2 qPCR F		TGT AGC TTC TCC CCC AAC AC	Amplification of *rrp2* from cDNA for expression studies
rrp2 qPCR R		TTT TGG CCA TGA AAA AGG AG	
bb0420QF		TGG CAA GTC GAA TAC CCT CT	Amplification of *hpk1* from cDNA for expression studies; From Rogers et al., 2009 [34].
bb0420QR		TGT TCG ATT TTA TTG GGA TGC	
bb0419F-RT		TTG AGG TTG CAA CAA ATG GA	Amplification of *rrp1* from cDNA for expression studies; From Rogers et al., 2009 [34].
bb0410R-RT		CGG GAT CGC TTT TTA GCT TT

**Table 1:** Bacterial strains and plasmids used in this study

Bacterial strain or plasmid	Genotype	Source
*Borrelia burgdorferi* B31A3	cp9^−^, wild type	Rocky Mountain Labs [59]
*E. coli* strains		
OneShot Top10	Cloning host; F^−^ *mcrA* Δ(*mrr-hsdRMS*-*mcrBC*) ϕ80*lacZ*ΔM15 Δ*lacX74 recA1 araD139* Δ(*ara leu*)*7697 galU galK rpsL*(Str^r^) *endA1 nupG*	Invitrogen
Rosetta (DE3)pLysS	Expression host; F^−^ *ompT hsdS*_B_(r_B_^−^ m_B_^−^) *gal dcm* (DE3) pLysSRARE (Cam^R^)	Novagen
Plasmids		
pCS1–5	pCR2.1(*bb0173*_*T*_)	This study
pCS1–9	pET23a(*bb0173*_*T*_)	This study

To evaluate the potential contributions of the Bat domains in BB0173 and BB0175, cultures were grown to late log phase under microaerophilic (standard oxygen with 1% CO_2_) or low oxygen conditions, and transcripts of *bb0170* to *bb0176* were analyzed. Low oxygen conditions were generated by including Oxyrase® to broth. Expression from genes *bb0170* to *bb0176* was evaluated using quantitative PCR (qPCR). Primers for *bb0170* to *bb0176* and control (constitutive expressing) gene *flaB* are described in Table 2. All reactions were repeated in triplicates, and three biological replicates were evaluated. Samples were amplified using PowerUP™ SYBR® Green (Applied Biosystems, Foster City, CA) following the manufacturer’s recommendations. Samples were amplified using the Bio-Rad CFX96 Touch™ Real-Time PCR Detection System (Table 2).

### Statistical analysis of gene expression data

The raw *bb0170* to *bb0176* threshold values were normalized to the reference gene flagellin (*flaB*) using the comparative C_T_ method [50, 51]. After normalization, the fold change was evaluated between the aerobic and low-oxygen grown samples. Upon determination of the fold change for each gene, the fold changes were evaluated for statistical significance using unpaired *t-*tests with the Holm-Sidak method of correction for multiple comparisons. In each case, a significance level of 0.05 was used. All analyses and expression graphics were performed using GraphPad Prism Version 6.0d.

### Computer-assisted analysis of BB0173 transmembrane regions

Putative insertion of hydrophobic regions (HR) from BB0173 proteins was predicted using the ΔG Prediction Server v1.0 using standard parameters combined with subsequent detection of the lowest apparent free energy differences (Δ*G*_app_ values) ([27]; http://dgpred.cbr.su.se/). Models of tertiary structure were generated using template 4jdu.1.A, a VWFA and BatA domain containing protein of *Ba. fragilis* (BF9343_2419) that is highly similar to BB0173, using SWISS-MODEL [52–54].

### Cloning of putative transmembrane regions

For the membrane insertion of isolated BB0173-segments, HR1 (residues 7–25), HR2 (residues 55–77), HR3 (residues 163–185) and HR4 (residues 310–328) fragments were independently amplified and introduced into the modified *E. coli* leader peptidase (Lep) sequence from the pGEM1 plasmid [28] using the *Spe*I/*Kpn*I sites. After an overnight ligation, constructs were electroporated into TOP10 cells. Positive clones were selected on ampicillin plates (100 μg/ml) and verified by sequencing (Eton Biosciences, San Diego, CA). After sequencing confirmation, clones found to be in frame with Lep protein were selected for use in the in vitro transcription-translation experiments.

Alternatively, we prepared templates for the in vitro transcription of the truncated *BB0173* mRNA with a 3′-glycosylation tag. BB0173 truncated constructs were obtained by using forward primers that include the T7 promoter sequence at the 5′ end. The 3′ reverse primers were designed to have approximately the same annealing temperature as the 5` forward primer, contained an optimized glycosylation C-terminal tag followed by tandem translational stop codons, TAG and TAA, and annealed at specific positions to obtain the desired polypeptide length as previously described [30]. Primers are described in Table 2.

Agarose gels (2%) were used to verify PCR product size then samples were cleaned using the Wizard® SV Gel and PCR Clean-up System (Promega, Madison, WI) following the manufacturer’s recommendations.

### In vitro transcription-translation

The BB0173 Lep-derived constructs and BB0173 truncated constructs were transcribed and translated using the TNT T7 Quick Coupled System (Promega, Madison, WI). The reactions contained 75 ng of DNA template, 0.5 μl of [^35^S]Met (5μC_i_), and 0.25 μl of microsomes (tRNA Probes) were incubated for 90 min at 30 °C. The translation products were ultracentrifuged (100,000 *g* for 15 min) on a sucrose cushion, and analyzed by SDS-PAGE. The bands were quantified using a Fuji FLA-3000 phosphoimager and Image Reader 8.1j software.

For the proteinase K protection assay, 2 μl of proteinase K (1 mg/ml) was added to the sample, and the digestion reaction was incubated for 15 min on ice. Before SDS-PAGE analysis, the reaction was stopped by adding 1 mM phenylmethanesulfonyl fluoride (PMSF).

For EndoH (New England Biolabs, Beverly, MA) treatment, 1 μl of 10X Glycoprotein Denaturing Buffer, 1 μl of 10X GlycoBuffer, 1 μl of EndoH and 7 μl of H_2_0 were added to make a 10 μl total reaction volume and incubated for 1 h at 37 °C with 0.1 mU of EndoH. The samples were analyzed by SDS-PAGE.

### Expression and purification of rBB0173_T_

The first hydrophobic regions of the BB0173 protein were excluded during cloning, in order to enhance the ability and viability of *E. coli* cells used to express *bb0173*. This N-terminally truncated version of *bb0173* will be referenced in this paper as rBB0173_T_ (Fig. 1b). The construct was generated using primers summarized in Table 2, which amplified the gene from *B. burgdorferi* B31A3 total genomic DNA as well as introduced restriction enzyme sites *Nde*I (5′) and *Xho*I (3′) prior to insertion into the conventional cloning vector pCR 2.1 – TOPO™ (Invitrogen™ LifeTechnologies®) following manufacturer’s recommendations. Positive clones were confirmed by sequencing (Eton Biosciences, San Diego, CA, USA) and sub-cloned into the expression vector pET23a (Novagen, Madison, WI) using *Xho*I and *Nde*I restriction enzymes engineered flanking the *bb0173*_*T*_ sequence as previously described [17]. The plasmid constructs containing inserts of expected sizes were sequenced and used to transform the *E. coli* expression host.

Truncated recombinant BB0173 (rBB0173_T_) with a C-terminal 6 × histidine tag was overexpressed by inducing the *E. coli* strain containing pET23a-*bb0173*_*T*_ with 1 mM IPTG for 3 h and purified following protocols previously developed [17]. Fractions with the highest concentration of rBB0173_T_ were combined and dialyzed against a buffer consisting of 50 mM sodium phosphate and 300 mM NaCl (pH 7.4; Slide-A Lyzer™ G2 dialysis cassette; Thermo Scientific, Waltham, MA) prior application to Amicon® Centrifugal Filters (EMD Millipore, Billerica, MA) to concentrate the protein. Quantification of protein concentration was achieved using a bicinchoninic acid (BCA; Pierce/Thermo Scientific, Waltham, MA) assay. Aliquots of the protein were stored at −80 °C until further use.

### Generation and purification of polyclonal antibodies against BB0173

Antibodies against BB0173 were generated to detect this *Borrelial* protein in immunoblot assays. Chickens were utilized as model for the generation of specific antibodies, and were housed at the Texas A&M University poultry farm. A 30-amino acid peptide derived from BB0173 (BB0173_pep_, amino acids 105–134) was generated (Peptide 2.0 Inc., Chantilly, VA) from the region found within the large loop predicted to contain the VWFA domain and just beyond the predicted Metal Ion Dependent Adhesion Site (MIDAS) motif (Fig. 1b). BB0173_pep_, sequence GAVEFSSKNRLEFSKELIRGFISQRENDNI, was rehydrated using 50% ethanol and was used to immunize chickens in parallel with rBB0173_T_ in order to compare antibody response, sensitivity, and specificity. Each hen (*n* = 3 per antigen) received 50 μg of either the truncated protein or the peptide using equal parts protein and TiterMax™ Gold adjuvant (Sigma-Aldrich, St. Louis, MO). Chickens were immunized intramuscularly through the breast at days 0, 14, and 28 to allow for the generation of a sufficient memory antibody response. From days 35 to 45, eggs were collected daily and frozen at −20 °C until antibodies were purified from the yolk as previously described [55]. Antibodies were determined to recognize both the truncated antigen as well as full length BB0173 in *Borrelial* whole cell lysates via ELISA and western blot. For this study, detection was carried out using BB0173_pep_ specific antibodies due to detection with lower background.

### Protease treatment of *B. burgdorferi*

Proteinase K degradation of proteins exposed to the extracellular environment was conducted using *B. burgdorferi* cells grown at 32 °C in BSK II pH 7.6 following previously standardized protocols [17]. Briefly, *B. burgdroferi* B31 A3 strain cells were washed in Hank’s Balanced Salt Solution (HBSS) containing 5 mM MgCl_2_ and 50 mM sucrose to enhance membrane stabilization. After washing, a whole cell lysate aliquot was separated, washed further, and stored at −20 °C until use. The rest of the cells were then treated with 0, 10, 20, 50, 100, or 200 μg Proteinase K and incubated at 37 °C for 30 min. After incubation, PMSF was added to each sample at a final concentration of 1 mM to stop Proteinase K activity. Cells were then washed in supplemented HBSS containing 1 mM PMSF. Treated cells were stored at −20 °C until use.

### Detergent phase partitioning

Triton X-114 phase partitioning was conducted using *B. burgdorferi* B31 A3 grown at 32 °C in BSK II pH 7.6, pelleted and washed in HBSS. Cells were treated with 1% Triton X-114 in HBSS and incubated at 4 °C overnight with gentle agitation. Cells were then centrifuged at 8000 x *g* and the pellet containing the protoplasmic cylinders (PC) was saved. The supernatant was treated with 2% then 10% Triton X-114. The detergent (DT) and aqueous (AQ) phases were washed in HBSS then precipitated with 10-fold volume of ice cold acetone, stored at −20 °C overnight and pelleted as previously described [56]. The supernatant was discarded and samples were stored at −20 °C until use.

### Protein resolution and detection

Both Triton X-114 and Proteinase K treated samples were analyzed using SDS-PAGE and immunoblot analyses. In both cases, SDS—12% PAGE gels were used to separate proteins from treated or untreated whole cell lysates from *B. burgdorferi*. After protein separation, gels were either stained or transferred to membranes for immunoblot. Gels for visualization were treated with either Coomassie brilliant blue in the case of Proteinase K treatment or Silver Stain Plus (Bio-Rad Laboratories, Inc., Hercules, CA) for Triton X-114 treated samples. For immunoblot analysis, gels were transferred to PVDF membranes (Hybond-P; GE Healthcare, Piscataway, NJ) as previously described [17]. The PVDF membranes were blocked overnight at 4 °C in Tris-buffered saline containing 0.2% Tween 20 (TBS-T) and 10% skim milk. After blocking, membranes were probed with chicken anti-BB0173_pep._ Primary control antibodies included: OMPs OspC and P66, and the cytosolic proteins superoxide dismutase A (SodA, cytosolic), oxidative stress regulator (BosR, periplasmic), and Flagellin B (FlaB, periplasmic) [46]. OspC, VlsE, and P66 were utilized to determine proteinase K activity on outer membrane proteins, while BosR and FlaB served intracellular controls. OspC (OMP) and FlaB (periplasmic) were used to as controls for the Triton X-114 detergent phase separation assay [57]. Blots were developed following incubation with appropriate dilutions of HRP-conjugated secondary antibodies and detected using ECL western blotting reagents (GE Healthcare, Piscataway, NJ) as previously described [46, 47, 57, 58]. All gels and blots were imaged using a ChemiDoc™ Touch Imaging System (Bio-Rad Laboratories, Inc. Hercules, CA).

## Abbreviations

AAA: ATPase Associated with diverse cellular Activities
AQ: Aqueous phase
Bat: Bacterioides aerotolerance
BCA: Bicinchroninic acid
BosR: Borrelia oxidative stress Regulator
BSK-II: Barbour-Stoenner-Kelly medium
*cmdA-D*: complex media growth deficient operon
CoADR: Coenzyme A Disulfide Reductase
CoASH: Coenzyme A
Ct: Carboxyl terminal
DT: Detergent phase
ECM: Extracellular Matrix
ELISA: EnzymeLinked Immuno-Sorbent Assay
*flaB*: Flageline B gene
G1 and G2: Glycosilation sites
HBSS: Hans Balance Salt Solution buffer
HR: Hydrophobic region
HRP: Horseradish peroxidase
iNRS: Inactivated Normal Rabbit Serum
LD: Lyme Disease
Lep: Protein leader peptide
MIDAS: Metal Ion Dependent Adhesion Site
MoxR: Methanol dehydrogenase regulatory ATPase
NapA: Neutrophil activating protein A
Nt: Amino terminal
OMP: Outer Membrane Proteins
OspC: Outer surface protein C
PC: Protoplasmis cylinders
PK: Proteinase K
PMSF: Phenylmethane sulfonyl fluoride
qPCR: Quantitative Polymerase Chain Reaction
SodA: Superoxide Dismutase A
TBS-T: Tris Buffer Saline –Tween 20
TM: Transmembrane domain
TPR: Tetratricopeptide
VlsE: Variable large protein E (antigenic variation)
VWFA: von Willebrand Factor A domain
ΔG: Gibbs free energy change
